# Changes in energy expenditure associated with ingestion of high protein, high fat versus high protein, low fat meals among underweight, normal weight, and overweight females

**DOI:** 10.1186/1475-2891-6-40

**Published:** 2007-11-12

**Authors:** Amy Jo Riggs, Barry D White, Sareen S Gropper

**Affiliations:** 1 Department of Health and Kinesiology, Georgia Southern University, Statesboro, Georgia, USA; 2 Department of Nutrition and Food Science, Auburn University, Auburn, Alabama, USA

## Abstract

**Background:**

Metabolic rate is known to rise above basal levels after eating, especially following protein consumption. Yet, this postprandial rise in metabolism appears to vary among individuals. This study examined changes in energy expenditure in response to ingestion of a high protein, high fat (HPHF) meal versus an isocaloric high protein, low fat (HPLF) meal in underweight, normal weight, or overweight females (n = 21) aged 19–28 years.

**Methods:**

Energy expenditure, measured using indirect calorimetry, was assessed before and every 30 minutes for 3.5 hours following consumption of the meals on two separate occasions. Height and weight were measured using standard techniques. Body composition was measured using bioelectrical impedance analysis.

**Results:**

Significant positive correlations were found between body mass index (BMI) and baseline metabolic rate (MR) (r = 0.539; p = 0.017), between body weight and baseline MR (r = 0.567; p = 0.011), between BMI and average total change in MR (r = 0.591; p = 0.008), and between body weight and average total change in MR (r = 0.464; p = 0.045). Metabolic rate (kcal/min) was significantly higher in the overweight group than the normal weight group, which was significantly higher than the underweight group across all times and treatments. However, when metabolic rate was expressed per kg fat free mass (ffm), no significant difference was found in postprandial energy expenditure between the overweight and normal groups. Changes in MR (kcal/min and kcal/min/kg ffm) from the baseline rate did not significantly differ in the underweight (n = 3) or in the overweight subjects (n = 5) following consumption of either meal at any time. Changes in MR (kcal/min and kcal/min/kg ffm) from baseline were significantly higher in normal weight subjects (n = 11) across all times following consumption of the HPHF meal versus the HPLF meal.

**Conclusion:**

There is no diet-induced thermogenic advantage between the HPHF and HPLF meals in overweight and underweight subjects. In contrast, in normal weight subjects, ingestion of a HPHF meal significantly increases MR (69.3 kcal/3.5 hr) versus consumption of a HPLF meal and provides a short-term metabolic advantage.

## Background

Obesity is a growing epidemic. Nearly two-thirds of American adults (65%) are overweight with one-third being obese (30%) and this number continues to climb [1]. Obesity accounts for thousands of deaths per year and on average costs $75 billion dollars per year in health care in the United States [2].

While the causes of obesity are multifactoral, weight gain ultimately results from an imbalance between energy intake and energy expenditure. The main components of energy expenditure are basal/resting needs and physical activity. Diet-induced thermogenesis (the rise in resting energy expenditure associated with food ingestion) is only a minor component representing on average about 10% of basal needs. Yet, the contribution of diet-induced thermogenesis can be much higher if high protein diets are consumed. Metabolic rate may increase up to about 30% after protein consumption [3].

High protein diets, such as the Atkins™ diet and Zone™ diet are popular for weight loss in the United States. For example, a 2002 Consumer's Report survey found that over 30% of dieters reported the use of the Atkins™ diet to help them lose weight [4]. Yet, while high protein diets provide good satiety [5,6] and are popular with the public, health professionals are generally more cautious about such diets. High protein diets often result in high fat consumption and may also promote increased calcium excretion and longer term problems such as an increased risk of heart disease, osteoporosis, kidney problems, and increased mortality [5,7,8].

Whether high protein diets offer a metabolic advantage through increased diet-induced thermogenesis over other diets that are lower in protein is not clear, since the thermogenic response to food is only one small component of energy expenditure and thus weight balance. Also unclear is whether there are differences in the thermogenic response to different nutrients in individuals of various body sizes. Should differences in responses exist among individuals, these differences over time could promote weight gain or loss.

To date, most studies have examined differences in diet-induced thermogenesis between normal weight and obese individuals with some studies showing no differences and others finding a diminished response in obese adults [9-13]. Few studies, however, have included healthy underweight individuals [14-16]. Further, while the macronutrient composition has varied considerably between studies, no studies have used a macronutrient composition similar to those found in the Atkins™ and Zone™ high protein diets. Because the use of these fad diets is common for weight control, the purpose of this study was to examine short-term changes in energy expenditure in response to ingestion of a high protein, high fat meal (designed by the Atkins Company) versus an isocaloric high protein, low fat meal (designed by the makers of the Zone diet) among healthy underweight, normal weight, and overweight/obese females.

## Methods

### Subjects

Subjects, recruited through posted flyers and oral announcements in nutrition and food science classes, consisted of 21 females aged 19–28 years. Subjects had to be healthy, free from nut and chocolate allergies, and have regular menstrual cycles to participate. The study was approved by the Auburn University Institutional Review Board for the Use of Human Subjects in Research.

### Study design

Subject participation required three meetings. At the first meeting, height and weight were measured using a stadiometer and calibrated beam scale (Detecto Medical Scale, Webb City, MO). From the height and weight measurements, each subject's body mass index (BMI) was calculated, and each subject was classified as underweight (<18.5 kg/m^2^), normal weight (18.5–24.9 kg/m^2^), or overweight (≥ 25.0 kg/m^2^) based on Centers for Disease Control criteria [17]. Body composition (fat free mass and body fat) was measured using bioelectrical impedance analysis (Bodystat Ltd., Tampa, FL). Subjects completed a consent form and medical questionnaire, and received a log to track the first and last day of their menstrual cycle.

Subjects reported for the second and third meetings for the meal trials at the same time of the month based on their menstrual cycle. In addition, subjects must have refrained from eating or drinking (except water) for 12 hours, refrained from caffeine use and exercise for at least 12 hours, and refrained from smoking the morning of their trial. Energy expenditure, assessed using indirect calorimetry (MedGem, Healthetech, Golden, CO), was measured at baseline between about 7 and 8 am after subjects sat quietly for 10 minutes. The term baseline rather than resting is being used since subjects drove to the testing site. Energy expenditure measurements took about 10 minutes/measurement and were conducted with subjects in a seated position in a darkened room with a room temperature of 72°F. Subjects were then given 15–20 minutes to consume a high protein (34% kcal, 37 g protein), high fat (43% kcal, 21.2 g fat) meal (2 Atkins™ Advantage bars, Atkins™ Nutritionals, Inc., Ronkonkoma, NY) or a high protein (28% kcal, 30.8 g protein), low fat (24% kcal, 11.8 g fat) meal (2 OmegaZone™ bars, Sears Labs, Inc., Marblehead, MA). Bars were analyzed for macronutrient contents by proximate analysis (Silliker, Inc., Chicago Heights, IL). Subjects received the two different meals, each providing 440 kcal, in random order on two different occasions, and the researcher was blinded to what bars the subjects received on each of the two visits. Following consumption, energy expenditure was measured every 30 minutes for 3.5 hours. During the time period following meal consumption and in between energy expenditure measurements, subjects were allowed to read or study in a seated position, were allowed one bathroom break per hour, and were allowed to drink water as desired.

### Statistical analysis

Statistical analyses were conducted using InStat (GraphPad Software, San Diego, CA) and JMP IN (SAS Institute, Thomson Learning, Belmont, CA) programs. The Restricted or Residual Maximum Likelihood (REML) method was used to determine the effects of BMI classification (overweight, normal weight, and underweight), meal type (high protein, low fat and high protein, high fat), time, and their interactions on energy expenditure. The effect of subject across time was also included in the model with random effects assigned to subjects. The potential effect of meal order was also determined. Differences between group means were determined by either Least Square Means Differences Student's t or orthogonal contrasts. Pearson correlations were used to assess the relationships between BMI and energy expenditure, and between body weight and energy expenditure.

## Results

Twenty-one females (1 Asian, 2 African Americans, and 18 Caucasians) aged 19–28 years participated in the study. Two subjects, however, only completed one trial each. Four other subjects completed body composition measurements in the initial visit, but failed to return. The main reason cited for not completing the study was the lack of time to commit to the study. In addition, a few underweight subjects were not eligible to participate due to abnormal menstrual cycles and/or disordered eating.

Mean (± SE) age, height, weight, BMI, fat free mass, and body fat for subjects are shown in Table 1. Of the 21 participants, six were overweight/obese (4 Caucasian, 2 African American), 12 were normal weight (11 Caucasian, 1 Asian), and three were underweight (3 Caucasian). BMI of the subjects ranged from 17.7 to 31.4 kg/m^2^. No significant differences were found among the three groups in age, height, or fat free mass, while body fat was significantly greater in the overweight group versus the normal weight and underweight groups. Most subjects (62%) completed the two meal trials within a week time frame; the remaining subjects (38%) completed the two meal trials within 1 to 2.5 months. No effect of meal order was found, and thus, it was not included in the model. The coefficient of variation in metabolic rate for subjects consuming the same diet on different occasions was 2.74%.

**Table 1:** Mean (± SE) age, height, weight, body mass index (BMI), fat free mass (FFM), and body fat among overweight, normal weight, and underweight subjects

Weight Classification			
	Overweight n = 6	Normal Weight n = 12	Underweight n = 3

Variable	Mean ± SE	Mean ± SE	Mean ± SE

Age (years)	22.83 ± 1.09^a^	20.75 ± 0.77^a^	20.67 ± 1.54^a^
Height (m)	1.62 ± 0.02^a^	1.65 ± 0.02^a^	1.69 ± 0.04^a^
Weight (kg)	70.77 ± 2.60^a^	58.08 ± 1.84^b^	51.72 ± 3.68^b^
BMI (kg/m2)	26.85 ± 0.74^a^	21.14 ± 0.52^b^	18.07 ± 1.04^c^
FFM (kg)	48.37 ± 1.74^a^	44.59 ± 1.22^a^	41.73 ± 2.83^a^
Body fat (%)	31.40 ± 1.22^a^	23.03 ± 0.86^b^	19.50 ± 1.72^b^

Note: ^abc^Values in a row with different superscripts differ significantly (p < 0.05).

**Figure 1:**
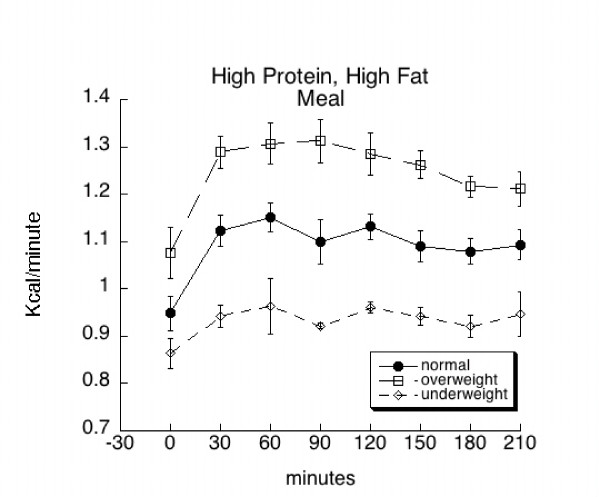
Mean (± SE) baseline and postprandial metabolic rate (kcal/min) among overweight, normal weight, and underweight subjects before and following consumption of a high protein, high fat (HPHF) meal.

**Figure 2:**
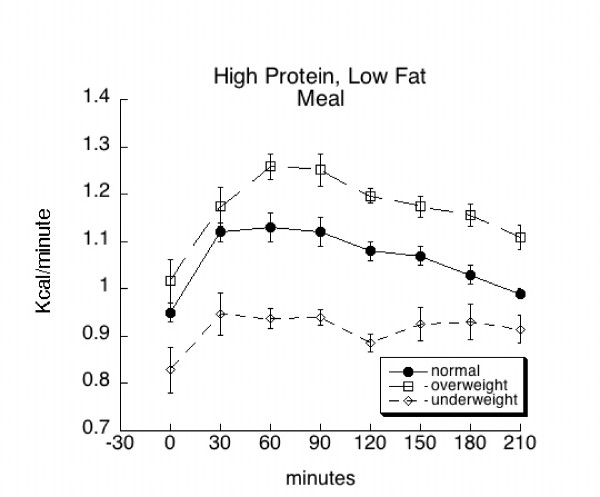
Mean (± SE) baseline and postprandial metabolic rate (kcal/min) among overweight, normal weight, and underweight subjects before and following consumption of a high protein, low fat (HPLF) meal.

There was a significant (p = 0.0003) effect of BMI classification on absolute (kcal/min) energy expenditure. This was true prior to (at baseline) and after consumption of the meals (Figures 1 and 2). Overall, individuals classified as overweight (1.20 ± 0.01 kcal/min) showed a greater level of energy expenditure as compared to individuals classified as normal weight (1.08 ± 0.01 kcal/min). Likewise, normal weight individuals showed greater energy expenditure than underweight individuals (0.92 ± 0.02 kcal/min) across all times and treatments. When energy expenditure was expressed as kcal/min/ffm, there was no difference among groups at baseline, but a significant (p = 0.0001) effect of BMI classification across all postprandial times and treatments (Table 2). However, when expressed in this manner (representing average energy expenditure over 210 minutes across both treatments), the difference in energy expenditure between overweight (0.025 ± 0.0003 kcal/min/kg ffm) and normal weight groups (0.024 ± 0.0002 kcal/min/kg ffm) was corrected. The energy expenditure of underweight group 0.022 ± 0.0005 kcal/min/kg ffm) was still significantly less than the other two groups across all times and treatments.

**Table 2:** Comparisons of mean (± SE) baseline and postprandial metabolic rate (kcal/min) expressed per kg of fat free mass (ffm) among groups

Metabolic Rate*						
	Overweight		Normal Weight		Underweight	

Time (min)	kcal/min/ffm		kcal/min/ffm		kcal/min/ffm	

	HPHF	HPLF	HPHF	HPLF	HPHF	HPLF
Baseline	0.022 ± 0.001	0.021 ± 0.001	0.021± 0.001	0.022 ± 0.001	0.021 ± 0.001	0.020 ± 0.001
30	0.026 ± 0.001	0.024 ± 0.001	0.025 ± 0.001	0.026 ± 0.001	0.023 ± 0.001	0.023 ± 0.001
60	0.027 ± 0.001	0.026 ± 0.001	0.026 ± 0.001	0.026 ± 0.001	0.023 ± 0.001	0.023 ± 0.001
90	0.027 ± 0.001	0.026 ± 0.001	0.025 ± 0.001	0.026 ± 0.001	0.022 ± 0.001	0.023 ± 0.001
120	0.027 ± 0.001	0.025 ± 0.001	0.025 ± 0.001	0.025 ± 0.001	0.023 ± 0.001	0.021 ± 0.001
150	0.026 ± 0.001	0.024 ± 0.001	0.024 ± 0.001	0.024 ± 0.001	0.023 ± 0.001	0.022 ± 0.001
180	0.025 ± 0.001	0.024 ± 0.001	0.024 ± 0.001	0.023 ± 0.001	0.022 ± 0.001	0.023 ± 0.001
210	0.026 ± 0.001	0.023 ± 0.001	0.025 ± 0.001	0.023 ± 0.001	0.023 ± 0.001	0.022 ± 0.001

* No difference among groups at baseline, but a significant (p = 0.0001) effect of BMI classification across all postprandial times and treatments. However, the difference in energy expenditure between the overweight and normal weight groups was not significant, but the energy expenditure of underweight group was still significantly less than the other two groups across all times and treatments.

There was a significant (p < 0.0001) effect of time on absolute energy expenditure irrespective of BMI and treatment (meal type). Overall, energy expenditure significantly increased by 30 minutes after consumption of the meal. The increased level of energy expenditure remained until 180 minutes, when it began to decrease toward baseline. However, at 210 minutes post-consumption, energy expenditure was still significantly greater than baseline.

Significant positive correlations were found between BMI and baseline metabolic rate (r = 0.539; p = 0.017), and between body weight and baseline metabolic rate (r = 0.567; p = 0.011). Significant positive correlations also were found between BMI and average total change in metabolic rate (r = 0.591; p = 0.008), and between body weight and average total change in metabolic rate (r = 0.464; p = 0.045).

Table 3 reports mean (± SE) changes in metabolic rate among overweight, normal weight, and underweight subjects after consumption of high protein, high fat and high protein, low fat meals. Data are also reported as mean percent energy expended per kcal ingested. When expressed as the change in energy expenditure there was still a significant (p = 0.0003) overall effect of BMI classification. The overweight group (0.158 ± 0.011 kcal/min) had the greatest change in energy expenditure, following by the normal weight group (0.126 ± 0.008 kcal/min), and then the underweight group (0.077 ± 0.016 kcal/min). Similarly, when energy expenditure was expressed as the change in kcal/min/ffm, the overall effect of BMI was significant (p = 0.0034), with the values for the overweight group significantly greater than the normal weight group which was significantly greater than the underweight group.

**Table 3:** Mean (± SE) changes in metabolic rate (kcal/min) and mean (± SE) change in energy expenditure from baseline per kcal ingested (EE/I) in overweight, normal weight, and underweight subjects after consumption of a high protein, high fat (HPHF) meal versus after consumption of a high protein, low fat (HPLF) meal

Changes in Metabolic Rate						
	Overweight		Normal Weight		Underweight	

Time (min)	kcal/min/ffm		kcal/min/ffm		kcal/min/ffm	

	HPHF	HPLF	HPHF	HPLF	HPHF	HPLF
30	0.21 ± 0.04^a^*	0.20 ± 005^a°°^	0.19 ± 0.02^a^*	0.17 ± 0.02^a°°^	0.08 ± 0.01^a^*	0.12 ± 0.03^a°°^
60	0.23 ± 0.07^a^*	0.20 ± 005^a°°^	0.21 ± 0.02^a^*	0.18 ± 0.03^a°°^	0.10 ± 0.06^a^*	0.11 ± 0.03^a^*
90	0.24 ± 0.08^a^*	0.24 ± 0.06^a°°^	0.15 ± 0.03^a^*	0.17 ± 0.03^a°^	0.06 ± 0.03^a^*	0.11 ± 0.03^a°^
120	0.21 ± 0.09^a^*	0.19 ± 0.06^a°°^	0.20 ± 0.02^a^*	0.13 ± 0.02^b°°^	0.10 ± 0.04^a^*	0.06 ± 0.06^a°^
150	0.19 ± 0.04^a^*	0.18 ± 0.06^a°°^	0.16 ± 0.02^a^*	0.11 ± 0.01^a°^	0.08 ± 0.05^a^*	0.10 ± 0.01^a°^
180	0.14 ± 0.07^a^*	0.14 ± 0.05^a°^	0.15 ± 0.02^a^*	0.08 ± 0.02^b°°^	0.06 ± 0.01^a^*	0.10 ± 0.03^a°°^
210	0.13 ± 0.09^a^*	0.08 ± 0.07^a°^	0.16 ± 0.02^a^*	0.04 ± 0.02^b°°^	0.08 ± 0.02^a^*	0.0.9± 0.02^a°^
Total	1.35 ± 0.46^a^*	1.29 ± 0.38^a°°^	1.20 ± 0.06^a^*	0.87 ± 0.11^b°^	0.55 ± 0.17^a^*	0.68 ± 0.19^a°°^
EE/I^#^(%)	9.2 ± 1.7^a^	8.8 ± 1.6^a^	8.2 ± 1.1^a^	5.9 ± 1.1^a^	3.8 ± 2.2^a^	4.6 ± 2.1^a^

Note: ^ab^Values in a row with the same superscript did not differ significantly between the HPHF meal and the HPLF meal within a BMI group.

*Values in a row did not differ significantly among BMI groups following consumption of the HPHF meal.

°Values in a row did not differ significantly among BMI groups following consumption of the HPLF meal.

^#^EE/I (%) represents the average energy expenditure over 210 minutes divided by 440 kcal intake × 100 (9)

There was also a significant (p < 0.0001) effect of time when expressed as the change in energy expenditure. However, there was not a significant difference in the overall effect of meal type, when the data were expressed as the change in energy expenditure. There was a tendency for a BMI classification and meal type interaction (p = 0.084). While there was not an effect of meal type in the overweight or underweight groups, the change in energy expenditure was significantly (p = 0.0014) greater in the normal weight group following consumption of the high protein, high fat meal versus following consumption of the high protein, low fat meal. Similar significant (p = 0.0007) results were found when examining change in metabolic rate per kg ffm. The difference in the increase in metabolic rate after consumption of the high protein, high fat meal versus the high protein, low fat meal in the normal weight subjects represents about 69.3 kcal per 3.5 hours (1.20– 0.87 kcal/min × 210 min = 69.3 kcal).

## Discussion

High protein diets remain a popular choice for dieting among Americans. Yet, while high protein foods appeal to the taste buds of many individuals and can be part of a weight loss diet, high protein diets can vary tremendously in fat and carbohydrate contents and can be extremely unhealthy if excessively high in fat or extremely low in carbohydrate. These variations in the macronutrient composition of diets may influence diet-induced thermogenesis. Diet-induced thermogenesis also has been shown to differ among individuals of different body sizes. These thermogenic differences over time could lead to changes in energy balance and thus body weight. This study examined short-term changes in energy expenditure among overweight, normal weight, and underweight females after consumption of a high protein, high fat meal versus a high protein, low fat meal.

### Energy expenditure

The overweight group had a significantly higher resting absolute energy expenditure as compared to the normal weight and underweight groups. Several studies have examined differences in resting metabolic rate among individuals of different sizes. Similar to the present findings, James et al [18], Hoffmans et al [19], Ravussin et al [20], De Palo et al [11], Swaminathan et al [21] and Schutz et al [9] found absolute resting metabolic rates were significantly higher in obese individuals versus normal weight individuals. Bosy-Westphal et al [22] demonstrated that an underweight group of subjects had a significantly lower absolute metabolic rate than a normal weight group and an obese group. Only a couple of studies have calculated metabolic rate per kg fat free mass and have found mixed results. As in the studies by Kashiwazaki et al [23] and Ravussin et al [20], the results of this study found no significant difference in baseline metabolic rate per kg fat free mass among the BMI groups. Significant correlations in the present study were found between baseline metabolic rate and both BMI and body weight. These findings also are consistent with other studies, which have examined relationships between resting metabolic rate and body weight [20,23].

### Postprandial thermogenic responses to the meals

The increases in metabolic rate (time effect) that occurred after overweight, normal weight, and underweight subjects consumed the high protein, high fat and the high protein, low fat meals are consistent with changes in metabolic rate associated with diet-induced thermogenesis. Postprandial metabolic rate was significantly different among the BMI groups across all times and treatments with the highest rate in the overweight group, followed by the normal weight, and the underweight groups; however, when expressed per kg ffm, no difference was found between the overweight and the normal weight group, suggesting the difference in metabolic rate between these two groups related to greater fat free mass in the overweight group. It should be noted that even though fat free mass was not statistically greater in the overweight group, it was numerically greater. Moreover, while increases in metabolic rate were exhibited among all groups, the rise was not significantly different from baseline in the underweight subjects. Only one study has examined postprandial changes in metabolic rate in healthy, underweight females. While differences between baseline and postprandial measurements were not published, Scalfi et al [16] reported that underweight females had a significantly reduced postprandial thermogenesis versus normal weight females.

Several studies have examined postprandial metabolic rate in normal weight versus overweight/obese individuals. Results of such studies vary with a few reporting significantly higher metabolic rates in normal weight versus overweight/obese individuals [24,25] and a few finding the opposite [10,11,26,27]. A few studies also have found no significant differences in diet-induced thermogenesis (expressed as kcal/min, percent of energy intake, and/or kcal/min/kg body weight or ffm) between normal weight and obese individuals [10,26]. Differences in study findings likely result from differences in data expression and multiple other factors such as differences in energy intakes consumed by subjects within and/or between weight classes. Diet-induced thermogenesis is known to vary with energy ingested as well as macronutrient composition. Thus, if subject groups are fed less energy than other groups, causes of differences in thermogenic response would be unclear – resulting either, for example, from the fewer calories ingested or from a blunted thermogenic response to meal ingestion or a combination. Consequently, comparisons between the findings of published literature are difficult.

### Comparisons of change in metabolic responses to the ingestion of the high protein, high fat meal versus the high protein, low fat meal within groups

No significant difference in change in metabolic rate was observed when overweight or underweight subjects consumed the high protein, high fat meal versus the high protein, low fat meal. In contrast, changes in metabolic rate (kcal/min and kcal/min/kg ffm) were significantly higher in normal weight subjects across all times measured following consumption of the high protein, high fat meal versus the high protein, low fat meal. While several studies have examined postprandial metabolic rate in subjects of different sizes after consumption of two different meals, these studies did not examine thermogenic responses within a weight group between the two meals [9,11,26,28].

Reasons for the differential thermogenic response in the present study between normal weight and overweight/obese individuals to meals are unclear. Some studies have suggested that the thermogenic response to carbohydrate intake is blunted in overweight/obese individuals [26,29-31]. In addition, one study suggested that the thermogenic response to fat is blunted in overweight/obese individuals [21]. However, the results of other studies have shown no significant differences in thermogenic response to carbohydrates or fat between overweight/obese and normal weight individuals [10,26,27].

In the present study, the greater thermogenic response in the normal weight individuals after consumption of the high protein, high fat meal (containing 37 grams of protein) versus the high protein, low fat meal (containing 30.8 grams of protein) likely resulted from the higher protein content of the high protein, high fat meal since the thermogenic response to fat and carbohydrate is thought to be about equal [28,32]. The lack of this differential response to the two different meals in the overweight subjects may have resulted from a blunted response to the high fat (43%) content of the high protein, high fat meal [21]. A blunted (or lower rise) in metabolic rate associated with the high protein, high fat meal would decrease the differences observed in the overweight group when comparing metabolic rates between the two meals resulting in no statistically significant differences at the various times measured.

Underweight individuals, like the overweight individuals, showed no significant difference in thermogenic response to the ingestion of the two meals. Only one study has previously reported a reduced thermogenic response to meals in underweight females but did not compare differences in response between two meals [16]. The reduced response observed in this study in underweight females (although limited by sample size) may have resulted from decreases in either or both obligatory and facultative thermogenesis. Further studies should investigate both the hormonal and sympathetic nervous system responses to meals in healthy, underweight individuals.

## Conclusion

Changes in metabolic rate in response to high protein, high fat versus high protein, low fat meals do not differ in overweight and in underweight females and thus, there is no metabolic advantage in diet-induced thermogenesis between the two meals. In contrast, in normal weight subjects, ingestion of a high protein, high fat meal significantly increases metabolic rate (69.3 kcal/3.5 hr) versus consumption of a high protein, low fat meal and provides a short-term metabolic advantage. Whether the higher diet-induced thermogenesis would persist with continuous ingestion of a high protein, high fat diet in normal weight individuals and would lead to greater changes in body weight is not known and requires additional studies.

## Competing interests

The author(s) declare that they have no competing interests.

## Authors' contributions

AJR helped design the study, conducted the study, analyzed the data, and wrote the manuscript. BDW assisted with data analysis and figures in the manuscript. SSG contributed to the study design, data analysis, and writing of the manuscript. This research was financially supported in part by Malone-Zallen Research Scholar Fund and the Alabama Agricultural Experimental Station, Project 13-006. All authors read and approved the final manuscript.
